# Advances in the Development of Shape Similarity Methods and Their Application in Drug Discovery

**DOI:** 10.3389/fchem.2018.00315

**Published:** 2018-07-25

**Authors:** Ashutosh Kumar, Kam Y. J. Zhang

**Affiliations:** Laboratory for Structural Bioinformatics, Center for Biosystems Dynamics Research, RIKEN, Yokohama, Japan

**Keywords:** molecular similarity, virtual screening, shape similarity, drug discovery, gaussian overlay, spherical harmonics, 3D Zernike descriptors

## Abstract

Molecular similarity is a key concept in drug discovery. It is based on the assumption that structurally similar molecules frequently have similar properties. Assessment of similarity between small molecules has been highly effective in the discovery and development of various drugs. Especially, two-dimensional (2D) similarity approaches have been quite popular due to their simplicity, accuracy and efficiency. Recently, the focus has been shifted toward the development of methods involving the representation and comparison of three-dimensional (3D) conformation of small molecules. Among the 3D similarity methods, evaluation of shape similarity is now gaining attention for its application not only in virtual screening but also in molecular target prediction, drug repurposing and scaffold hopping. A wide range of methods have been developed to describe molecular shape and to determine the shape similarity between small molecules. The most widely used methods include atom distance-based methods, surface-based approaches such as spherical harmonics and 3D Zernike descriptors, atom-centered Gaussian overlay based representations. Several of these methods demonstrated excellent virtual screening performance not only retrospectively but also prospectively. In addition to methods assessing the similarity between small molecules, shape similarity approaches have been developed to compare shapes of protein structures and binding pockets. Additionally, shape comparisons between atomic models and 3D density maps allowed the fitting of atomic models into cryo-electron microscopy maps. This review aims to summarize the methodological advances in shape similarity assessment highlighting advantages, disadvantages and their application in drug discovery.

## Introduction

Molecular similarity is a key concept in drug discovery and has been routinely used in the discovery and design of new molecules. It is based on the notion that two molecules often share similar physical properties and biological function if they are structurally similar. This similarity principle has been widely utilized in early phases of drug development to discover new molecules. Virtual screening has been used to filter large databases of compounds to a smaller number based on this similarity principle. Molecular similarity has been also employed to optimize the potency and pharmacokinetic properties of lead compounds based on their structure–activity relationships.

There are two components of molecular similarity analysis (1) structural representations and (2) quantitative measurements of similarity between two structural representations. Many types of structural representations have been suggested to measure the similarity between two molecules. These include physiochemical properties, topological indices, molecular graphs, pharmacophore features, molecular shapes, molecular fields etc. Further, there are various methods to quantify the similarity between two structural representations, e.g., Tanimoto coefficient, Dice index, cosine coefficient, Euclidean distance, Tversky index etc. Among these, Tanimoto coefficient (Rogers and Tanimoto, 1960) is the most popular and widely used similarity measure. Based on the structural representation, molecular similarity approaches can be broadly classified into 2D or 3D similarity methods. The 2D similarity methods rely only on the 2D structural information and are among the fastest, efficient and most popular similarity search methods. Moreover, they do not rely on structural alignments for estimating the similarity between two molecules. These methods include substructure search, fingerprint similarity search and 2D descriptor-based methods. However, most of these methods are limited in their ability to enable scaffold hopping and provide no structural and mechanistic insights. To deal with the limitations associated with 2D similarity methods, several approaches were developed that account for 3D conformations of a molecule while performing similarity search. These methods include pharmacophore modeling, shape similarity, molecular field-based methods, 3D fingerprints among others. In recent years, ligand 3D shape-based similarity analysis has become a method of choice in increasing number of virtual screening campaigns. Several successful applications of shape similarity to discover new molecules have been published in the literature. The major advantage with shape-based virtual screening methods is that scaffold hopping can be conveniently accomplished and scaffolds other than the query can be identified.

In this review, we will summarize the development and application of various 3D shape similarity methods and will comment on their utility in drug discovery. We will first outline the classification and various types of 3D shape similarity methods highlighting their advantages and disadvantages. Later, we will describe various applications of 3D shape similarity methods in drug discovery.

## 3D shape similarity methods

**Figure 1:**
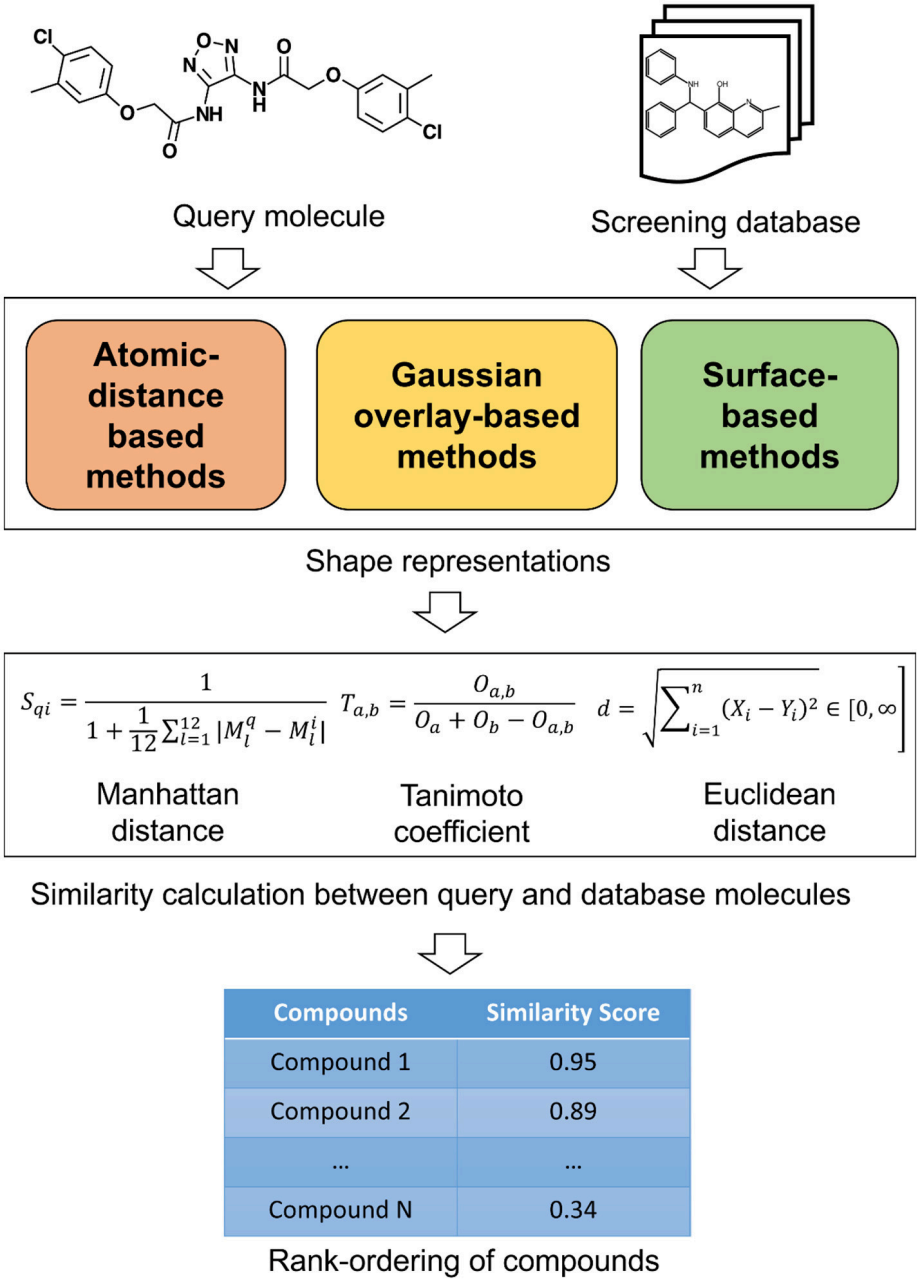
A schematic overview of similarity calculation between a query and database molecules.

The 3D shape has been widely recognized as a key determinant for the activity of small molecules and other biomolecules (Zauhar et al., 2003; Rush et al., 2005; Schnecke and Boström, 2006; Kortagere et al., 2009). The shape complementarity between ligand and receptor is necessary for bringing the receptor and ligand sufficiently close to each other so they can form critical interactions necessary for binding. Two molecules with similar shape are likely to fit the same binding pocket and thereby exhibiting similar biological activity. Shape comparison methods could be broadly classified as (1) Alignment-free or non-superposition methods and (2) Alignment or superposition-based methods. Both of these methods have their own advantages and disadvantages. Alignment-free methods are independent of the position and orientations of molecules. As such, they are much faster and could be used to screen large compound databases. Alignment-based methods rely on finding the optimal superposition between the compounds. Alignment-based methods are highly effective in identifying shape similarities among the molecular structures but they are computationally expensive. These methods enable comparison of the surface properties such as hydrophobicity and polarity. Visualization is one of the advantages with the alignment-based methods and the similarity between two molecules can be displayed. This information is useful in the design of new molecules and to guide further optimization. However, a subpar molecular alignment may lead to errors in comparing two molecules. Apart from this broad classification, shape similarity methods could be classified based on the underlying representation of molecular shape. The similarity between these shape representations is evaluated by employing various similarity metrics. A schematic overview of the similarity calculation between a query and database molecules is given in Figure 1. In the following paragraphs, we will outline commonly utilized shape representations with their advantages and disadvantages. As this review is targeted toward a broader readership, we will only provide an overview of the methods. For algorithmic details and mathematics behind each method, original publications may be referred.

### Atomic distance-based descriptors

These methods are based on the assumption that the shape of a molecule can be described by the relative positions of its atoms. The similarity between molecules can be then calculated by comparing the corresponding distributions of atomic distances. As these descriptors only require the computation of interatomic distances in compounds, these methods are faster compared to other shape comparison methodologies. Additionally, these methods do not require the alignment between two molecules for shape comparison. An overview of various atomic distance-based methods is given in Table 1 highlighting their availability as well as their advantages and disadvantages. One of the earlier atomic distance-based shape comparison method was based on atom triplet distances (Bemis and Kuntz, 1992). This method considered each molecule as a collection of three atom sub-molecules. The atom triplet triangle perimeters were used to generate shape histograms which were then utilized to compare the shape of molecules. This method however has a few limitations. It is difficult to select bin size suitable for all molecules. Each molecule typically generates 300–500 atom triplets and storing them require large space especially when comparing a large database of molecules. To deal with this limitation, another atom triplet based molecular shape comparison method was developed where a 2,048 bits long single condensed triplet shape signature was employed to represent the entire set of triplets in each molecule (Nilakantan et al., 1993). A signature of the query molecule is first compared with the already stored signatures of database molecules. Then only the compounds with adequately similar signatures are compared in detail by generating all triplets. Although this method was efficient but there was a risk of missing similar compounds due to the use of highly reduced signature representation. Another group developed molecular descriptors based on atom triplet triangles, angular information from surface point normal and local curvature to facilitate shape comparisons (Good et al., 1995). However, these descriptors have limited discriminating power and require large disk space for storage.

**Table 1:** Atomic distance based shape comparison methods.

**Method**	**Description**	**Availability**	**References**
USR	Extremely fast shape comparison method. Webserver can screen about 55 million conformers in 1 s. Different functional groups and enantiomers not recognized.	A ligand-based virtual screening webserver, USR-VS is available at http://usr.marseille.inserm.fr	Ballester and Richards, 2007a,b; Ballester, 2011; Li et al., 2016
USR+MACCS	Functional group information added to USR. Enantiomers not recognized.	Available on request	Cannon et al., 2008
CSR and USR:OptIso	Chiral shape recognition. Optical isomerism descriptors added to USR.	Developed by University of Oxford, UK. May be available from Oxford Drug Design company (https://www.oxforddrugdesign.com), Another implementation USR:OptIso is available at https://code.google.com/archive/p/usrchirality/	Armstrong et al., 2009; Zhou et al., 2010
Electroshape	Chiral shape recognition, include descriptor for charge and lipophilicity.	Developed by University of Oxford, UK. May be available from Oxford Drug Design company (https://www.oxforddrugdesign.com), A similarity search webserver including Electroshape implementation is available at http://www.swisssimilarity.ch	Armstrong et al., 2010, 2011; Zoete et al., 2016
UFSRAT	Pharmacophoric constraints by including atom-type information.	Developed by University of Edinburgh. Server available at http://opus.bch.ed.ac.uk/ufsrat/index.php	Shave, 2010; Lim et al., 2011; Shave et al., 2015
USRCAT	Included CREDO atom-type information.	A python implementation of the method using RDKit toolkits is available from https://bitbucket.org/aschreyer/usrcat	Schreyer and Blundell, 2009, 2012; Li et al., 2016
ACPC	Method uses autocorrelation of partial charges. High throughput virtual screening possible. Cannot distinguish a molecule from its enantiomer.	Developed by Laboratory for Structural Bioinformatics, Centre for Biosystems Dynamics Research, RIKEN and is available from http://www.riken.jp/zhangiru/software.html.	Berenger et al., 2014

**Figure 2:**
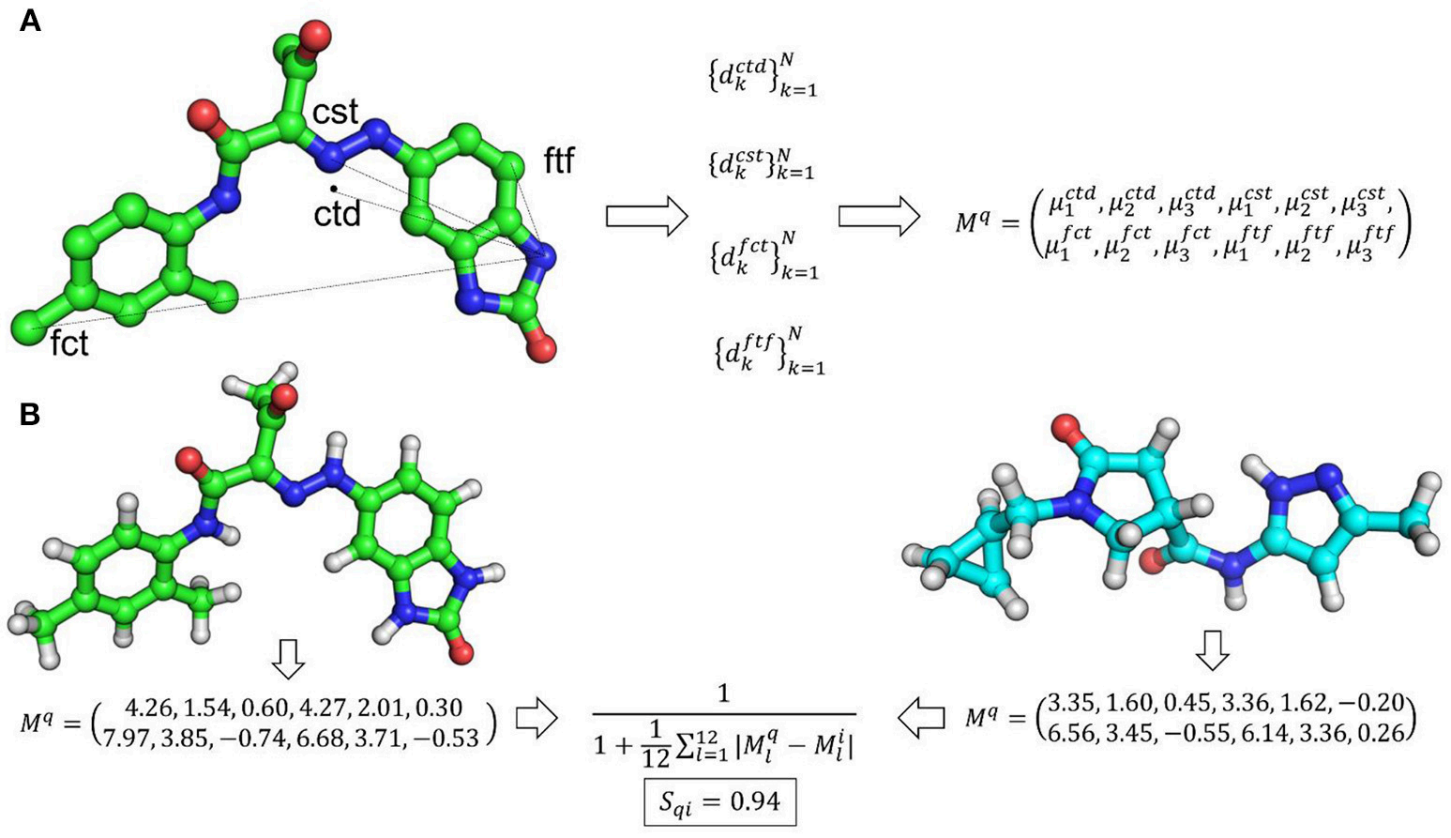
**(A)** An overview of USR shape representation. In USR approach, the shape of a molecule is described by the distribution of atomic distance to four reference points. **(B)** An example of shape similarity calculation between two small molecules utilizing the USR approach.

Ultrafast shape recognition (USR) (Ballester and Richards, 2007a,b; Ballester, 2011) is possibly the most popular atomic distance-based method developed to overcome alignment and speed problems associated with shape similarity methods. This method also uses the relative positions of atoms to describe the shape of a molecule. The schematic overview of USR method is given in Figure 2 along with an example of the shape similarity evaluation. USR calculates the distribution of all atom distances from four reference positions: the molecular centroid (ctd), the closest atom to molecular centroid (cst), the farthest atom from molecular centroid (fct) and the atom farthest away from fct (ftf). Consecutively, the first three statistical moments (mean, variance, and skewness of distribution) are calculated from each of these distributions. Hence, each molecule has a vector of twelve descriptors to describe its 3D shape. Finally, the similarity between shapes of two molecules is calculated through an inverse of the Manhattan distance of these 12 values:

$$\begin{array}{l} S_{qi}=\frac{1}{1+\frac{1}{12}\sum_{l=1}^{12}|M_{l}^{q}-M_{l}^{i}|} \end{array}$$

where *M*^*q*^ and *M*^*i*^ are vectors of shape descriptors for query and *i*^th^ molecule, respectively. The performance of USR was retrospectively compared with EigenSpectrum Shape Fingerprints (EShape3D) where better mean enrichment for USR was observed (Ballester et al., 2009). A retrospective comparison with three state-of-the-art shape similarity methods: EShape3D, shape signatures and ROCS revealed that USR is 1,546, 2,038, and 14,238 times faster than each one of them respectively (Ballester and Richards, 2007a). A web implementation of USR (USR-VS) is an extremely fast way of carrying out shape similarity calculations (Li et al., 2016). USR-VS is capable of screening 55 million 3D conformers per second and can calculate similarity scores for 94 million 3D conformers in about 2 s. This extremely fast speed is achieved as the features for all 3D conformers are preloaded into the memory. Moreover, the multi-threaded design of the webserver and alignment-free nature of USR method also contributed to such a high computational efficiency. A hardware implementation of USR has been shown to achieve two-fold speed gains over standard CPU based implementation of USR (Morro et al., 2018). In this implementation, a computing technique, Spiking Neural Networks, has been adapted utilizing Field-Programmable Gate arrays to allow highly parallelized implementation of USR. Prospective application of USR in the identification of arylamine *N*-acetyltransferases, protein arginine deiminase 4 (PAD4), falcipain 2, phosphatases of regenerating liver (PRL-3), p53-MDM2 inhibitors and for phenotypic targets such as colon cancer cell lines established the real-world applicability of USR (Li et al., 2009; Ballester et al., 2010, 2012; Teo et al., 2013; Hoeger et al., 2014; Patil et al., 2014). As USR is an ultrafast, purely shape-based similarity method, several methods augmenting the original USR capabilities were developed. These include a method where USR was combined with MACCS key encoding the topological information of small molecules (Cannon et al., 2008). To clearly distinguish between enantiomers, methods complementing USR with optical isomerism descriptors were developed (Armstrong et al., 2009; Zhou et al., 2010). Electroshape, a USR variant appended partial charge and atomic lipophilicity (alogP) as additional molecular properties to account for electrostatics and lipophilicity along with shape recognition (Armstrong et al., 2010, 2011). A web implementation of Electroshape is available at SwissSimilarity (Zoete et al., 2016). AutoCorrelation of Partial Charges (ACPC) also utilized partial charges with atomic distances to measure similarity between two molecules (Berenger et al., 2014). The method uses an autocorrelation function and a point charge model to encode all atoms of a molecule into two vectors that are rotation translation invariant. Another implementation of USR method is Ultrafast Shape Recognition with Atom Types (UFSRAT) which introduced pharmacophoric constraints to USR by incorporating atom type information (Shave, 2010; Lim et al., 2011; Shave et al., 2015). UFSRAT is capable of very fast comparison of query molecule with small molecule libraries from several major chemical vendors via its webserver (Table 1). Application of UFSRAT method in the discovery of MDM2, PRL-3, FK506-Binding Protein 12, kynurenine 3-monooxygenase and 11β-hydroxysteroid dehydrogenase type 1 (11βHSD1) inhibitors demonstrated its utility in key areas of drug discovery such as cancer, Alzheimer's disease, inflammation and type-II diabetes. (Hoeger et al., 2014; Houston et al., 2015; Shave et al., 2015, 2018). Another similar implementation, USRCAT utilized CREDO atom types to encode pharmacophoric information to USR (Schreyer and Blundell, 2009, 2012). USRCAT not only retained USR abilities to retrieve hits with low structural similarity but also demonstrated improved performance over the original USR implementation.

Atomic distance or descriptor-based methods are widely used due to their ability to quickly compare the shapes of query molecules with large small molecule libraries. A fast comparison of a wide range of chemical space increases the chances of finding novel hits. These methods are not only computationally efficient but also have produced excellent hit rates as revealed from several successful prospective studies against a wide range of molecular and non-molecular targets. Moreover, they are also capable of retrieving chemical scaffolds which are different from the query molecule, thus allowing scaffold hopping. As atomic distance-based shape similarity approaches are alignment-free, the visual inspection of shape similarity may be sometimes challenging especially for molecules which have low structural similarity. Selection of the right query compound is a key component of atomic distance-based shape similarity methods and their performance depends on optimal query selection. Hit rate can be improved by employing multiple queries and increasing the diversity of selected hits. Moreover, clustering based on shape similarity could be utilized to understand how different chemotypes arrange in binding pockets and thereby generating consensus queries (Pérez-Nueno et al., 2008; Pérez-Nueno and Ritchie, 2011) to improve virtual screening performance and reducing false positives.

### Atom-centered gaussian-based shape similarity methods

Among many methods of describing the molecular shape of a molecule, hard sphere (Connolly, 1985; Masek et al., 1993) and Gaussian sphere (Grant and Pickup, 1995; Grant et al., 1996) are two most widely adopted models. Both of these models describe the shape in terms of the volume of a molecule. Two molecules will possess similar shape if they have similar volume. Hard sphere model represents a molecule by a set of merged spheres where each sphere serves as an atom with its van der Waals radius. The volume of a molecule can be calculated by a formula that describes the union of a number of sets and their intersection. Although the analytical expression of the volume and its derivatives is reported in the original publication (Masek et al., 1993), it is not easy to implement as the formulas become very complicated with increasing number of intersections. Gaussian sphere model (Grant and Pickup, 1995, 1997; Grant et al., 1996) represents a molecule using a set of overlapping Gaussian spheres and measures the integral volume over all overlapping Gaussians. In this model, each intersection is expressed as the integral of a set of overlapping atom-centered Gaussian spheres and the volume of a molecule is described based on the inclusion-exclusion principle. Analytical expression for the volume calculation is given in the original publication which describes highly accurate volume calculation up to sixth order intersections (Grant and Pickup, 1995). The authors also proposed comparing shapes of two molecules by numerically optimizing the overlap between two molecules (Grant et al., 1996).

**Figure 3:**
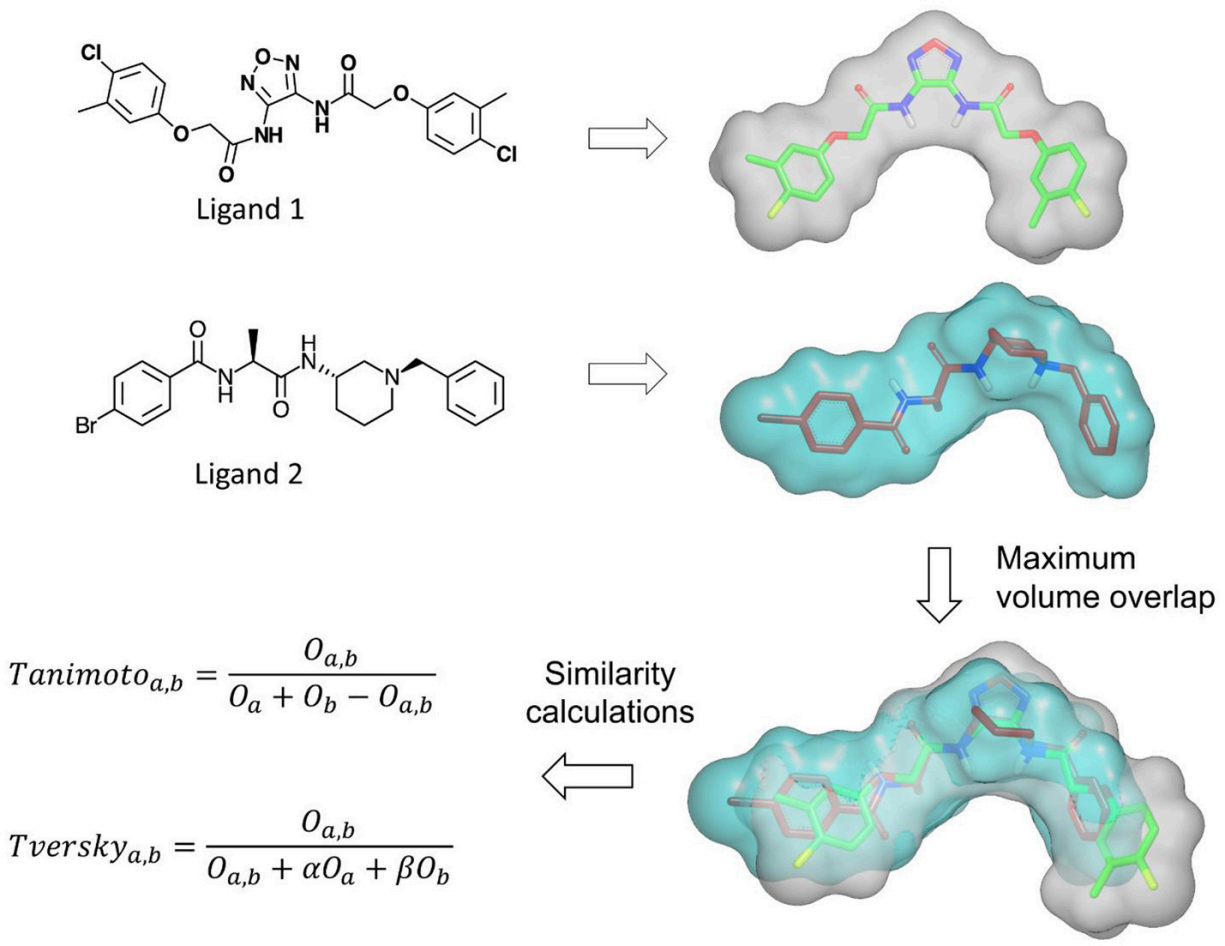
An overview of the shape similarity calculation by ROCS program.

Several methods based on Gaussian overlays were developed to measure the shape similarity between two molecules. An overview of these methods is presented in Table 2. Among these, Rapid Overlay of Chemical Structures (ROCS) is undoubtedly the most widely used method that utilizes Gaussian functions to measure the shape similarity between two molecules (Rush et al., 2005; Hawkins et al., 2007). ROCS algorithm is based on the original Gaussian overlay approach that finds and quantifies the maximum volume overlap between two molecules (Grant and Pickup, 1995; Grant et al., 1996). An overview of ROCS shape similarity calculation is given in Figure 3. However, to improve the efficiency of volume overlap calculations, it incorporated several modifications to the original implementation. ROCS ignores hydrogens for the volume calculations and uses equal radii for all heavy atoms. Furthermore, ROCS utilizes only the first order terms of shape density function. ROCS employs Tanimoto (Rogers and Tanimoto, 1960) and Tversky (Tversky, 1977) correlation coefficients as similarity metrics to calculate the overlap between two molecules which are defined as:

$$\begin{array}{l} Tanimoto_{a,b}=\frac{O_{a,b}}{O_{a}+O_{b}-O_{a,b}}\\ Tversky_{a,b}=\frac{O_{a,b}}{O_{a,b}+\alpha O_{a}+\beta O_{b}} \end{array}$$

where *O*_*a, b*_ is the volume overlap between molecules *a* and *b, O*_*a*_ is the volume of molecule *a* and *O*_*b*_ is the volume of molecule *b*. α and β are parameters for Tversky index. ROCS also considers chemical complementarity by including the chemical features to improve shape-based superposition. ROCS has been successfully employed in various drug discovery campaigns such as in the identification of small molecules inhibitors (Kumar et al., 2014b), to scaffold hop from one chemical class to another (Kumar et al., 2016), to rescore docking generated poses (Kumar and Zhang, 2016a) and to predict binding poses and ranking of inhibitors (Kumar and Zhang, 2016b,c). ROCS can routinely perform shape and chemical feature comparisons of about 600–800 conformers per second on a modern CPU. Although this speed is reasonable for alignment-based shape similarity methods, it takes several hours to screen a moderately sized virtual screening library. To facilitate large scale shape comparison, e.g., to screen large small molecule libraries within minutes, FastROCS (https://www.eyesopen.com/molecular-modeling-fastrocs), a GPU implementation of ROCS has been developed that increased the shape comparison speed by about three orders of magnitude over its CPU implementation. FastROCS is capable of processing up to a million conformers per second on a single NVIDIA Tesla K20 GPU (https://docs.eyesopen.com/toolkits/python/fastrocstk/architecture.html). PAPER, an open source GPU implementation of ROCS algorithm, also demonstrated speed acceleration up to two orders of magnitude on an NVIDIA GeForce GTX 280 GPU over its open source CPU implementation on a Intel Xeon E5345 CPU (Haque and Pande, 2010). MolShaCS is another method that engages Gaussian description of shape to evaluate molecular similarity between two molecules (Vaz de Lima and Nascimento, 2013). In addition to shape, MolShaCS utilizes Gaussian description of charge distribution to optimize overlays and similarity computations using Hodgkin's index (Hodgkin and Richards, 1987; Good et al., 1992). It was able to process 21 compounds per second, which seems to be a quite impressive speed for computers of that time. As Gaussian overlay based methods require precise alignment for the calculation of shape similarity, several groups employed approaches such as pharmacophore and field based methods to generate initial alignment. SHAFTS (*SHA*pe-*F*ea*T*ure *S*imilarity) (Liu et al., 2011) adopted pharmacophoric point triplets and least square fitting to generate initial alignment. A weighted sum of pharmacophoric fit and volume overlap was then used to assess shape similarities. Phase Shape (Sastry et al., 2011) also employed the same concept of atom distribution triplets to generate initial alignments which were then refined by maximizing the volume overlap. Phase Shape is capable of performing shape comparisons of about 500 conformers per second. Reminiscent of Shape and Electrostatic Potential (ShaEP) (Vainio et al., 2009) also resembles SHAFTS and Phase Shape as it utilizes a hybrid approach that combined field-based methods with volumetric methods to estimate molecular similarity. ShaEP borrowed a graph matching algorithm to generate initial superposition. Molecular graphs represented shape and electrostatic potential at points close to molecular surface. The method then optimized the initial alignment by maximizing the volume overlap calculated through Gaussian functions. Another similar method, SimG (Cai et al., 2013), adopted downhill simplex method (Nelder and Mead, 1965) to evaluate the similarity in shape and chemical features of a molecule and a binding pocket or ligand. SimG shape similarity method possessed advantage over other methods described here in the sense that it is capable of performing shape similarity evaluations between a ligand and a binding pocket. SABRE method (Hamza et al., 2012, 2013) introduced two modifications to the original Gaussian overlay based shape similarity implementation. First, it utilized reduced chemical structures by removing the functional group not present in query to generate initial alignments. Reduced chemical structures were subsequently replaced by full structures and the initial alignments were refined by rigid-body translation and rotation using steepest descent to produce shape density overlap with the query. Secondly, to avoid bias for large sized ligands when using Tanimoto similarity metric, a new scoring function Hamza–Wei–Zhan (HWZ) score was developed. An extension to SABRE method enabled its utility in chemogenomics area (Wei and Hamza, 2014). Shapelets (Proschak et al., 2008) is unlike any other Gaussian overlay based shape comparison method. It describes the shape of a molecule by decomposing its surface into discrete patches. This 3D graph representation can then be used for either full or partial shape similarity evaluations.

**Table 2:** An overview of commonly used Gaussian overlay based shape comparison methods.

**Method**	**Description**	**Availability**	**References**
ROCS	Fast Gaussian overlay based shape comparison. Widely used shape based virtual screening tool. GPU version also available.	Developed by OpenEye Scientific Software (https://www.eyesopen.com). Commercial.	Rush et al., 2005; Hawkins et al., 2007
PAPER	Accelerates large scale virtual screening experiments. Parallel implementation on NVIDIA GPUs.	Developed by Stanford University. Open source. Available from SimTK at https://simtk.org/projects/paper	Haque and Pande, 2010
MolShaCS	Uses Gaussian description of shape and charge. Hodgkin like similarity metric. Molecules are considered rigid.	Developed by University of Sao Paolo, Brazil. Open source tool available at https://code.google.com/archive/p/molshacs/downloads	Vaz de Lima and Nascimento, 2013
SHAFTS	It combines shape similarity with pharmacophoric features. Employs a hybrid similarity metric combining shape and chemical similarity. Suitable for large scale virtual screening.	Developed by Shanghai Key Laboratory of New Drug Design, East China University of Science & Technology, Shanghai, China. Available for download from http://lilab.ecust.edu.cn/home/resource.html	Liu et al., 2011
Phase Shape	Uses atom triplets to generate initial alignments which are refined by Gaussian overlay.	Developed by Schrodinger. (https://www.schrodinger.com). Commercial.	Sastry et al., 2011
ShaEP	Generate consensus shape pattern based on structural features of known ligands.	Developed by Abo Akademi University, Finland. Free for Academics. Available from the Abo Akademi University at http://users.abo.fi/mivainio/shaep/index.php	Vainio et al., 2009
SimG	Uses downhill simplex method to evaluate shape and chemical similarity between two molecules. Comparison of ligand and binding pocket shape or chemical similarity is also possible.	Developed by Shanghai Key Laboratory of New Drug Design, East China University of Science & Technology, Shanghai, China. Available for download from http://lilab.ecust.edu.cn/home/resource.html	Cai et al., 2013
SABRE	Uses consensus shapes to generate initial alignments which are later refined by rigid-body rotations and translations.	Academic license is available on request	Hamza et al., 2012, 2013
WEGA	Uses a weighted Gaussian function to improve the accuracy of first order approximation. A GPU implementation (gWEGA) is also available for large scale virtual screenings.	Developed by Research Center for Drug Discovery, Sun Yat-sen University, China. Academic license is available on request at http://www.rcdd.org.cn/home/program.html.	Yan et al., 2013

In most Gaussian function based overlay methods shape density of a molecule is described as the sum of shapes of individual atoms which sometimes results in the overestimation of the volume, for example, in molecules where some atoms highly overlap with others in the vicinity. Weighted Gaussian algorithm (WEGA) method (Yan et al., 2013) puts forward a modification where a weight factor is introduced for every atom. This weight factor reflects the crowdedness of an atom with its neighbors. The shape density of a molecule is represented by the linear combination of weighted atomic Gaussian functions. Utilizing this modification, WEGA method demonstrated improved shape similarity and virtual screening performance. The speed of WEGA shape similarity calculations varies with the size of query and database compounds. For an average drug-like query, WEGA can process 1,000–1,500 conformations per second (Yan et al., 2013). A GPU implementation of this method (gWEGA) has also been developed that reported a virtual screening speed increase by two orders of magnitude on one NVIDIA Tesla C2050 GPU over its CPU implementation on a quad-core Intel Xeon X3520 CPU (Yan et al., 2014). Another WEGA derivative, HybridSim proposed a hybrid metric combining 2D fingerprints with WEGA shape similarity and demonstrated improved virtual screening performance over standalone 2D fingerprint and shape similarity methods (Shang et al., 2017).

Overall, atom-centered Gaussian-based shape similarity methods present many advantages over other shape similarity methods. Although not as fast as distance based methods, these methods are fast enough for large scale virtual screenings. The major advantage with atom-centered Gaussian-based shape similarity methods is the visualization. The visualization of shape similarity between two molecules is immensely helpful in deriving the structure activity relationship for the optimization and for scaffold hopping. A majority of these methods address the problem of ligand flexibility by utilizing conformational ensemble. However, in some cases it may not be trivial to sample all possible conformations, e.g., natural products. Moreover, several top performing conformational generation methods face difficulty in modeling the correct conformation of some molecules, e.g., macrocycles, peptidomimetics etc. Another limitation with these methods is that their performance highly depends upon the query molecule and choosing the right query is a critical component of a shape-based virtual screening campaign (Kirchmair et al., 2009). Despite these limitations, atom-centered Gaussian overlay based methods are the most widely used shape similarity methods. They have provided many successful examples demonstrating their utility in various areas of drug discovery which will be discussed later in this manuscript.

### Surface based 3D shape similarity comparison methods

Molecular surface is another way of depicting the shape of a molecule. Comparison of molecular surfaces based on their shapes can reveal similarity in their physical and biological properties. There are many ways to describe the surface of a molecule. Precise definitions such as surface based on quantum mechanical wave functions are not practical especially for large molecules (Mezey, 2007). Surface definitions such as solvent-accessible surface (Lee and Richards, 1971; Connolly, 1983) and van der Waals surface are more practical and much easier to calculate. Some studies employed alpha shapes (Edelsbrunner et al., 1983; Edelsbrunner and Mücke, 1994; Edelsbrunner, 1995) which is a coarse representation of Connolly surface (Connolly, 1983) to describe the shape of a molecule (Wilson et al., 2009). Alpha shapes of a set of points “S” are generalization of convex hull and utilize a parameter, α to describe the shape with varying levels of details. For large α values, the alpha shape is equivalent to convex hull and shape feature details such as concavities and voids started to appear with decrease in α value. The alpha shape method has been applied to represent and compare shapes of 3D molecules (Wilson et al., 2009).

Shape signatures or shape histograms offer another representation of molecular shape that can be used to explore 3D volume of a molecule confined by the solvent accessible surface (Zauhar et al., 2003; Meek et al., 2006). Shape signatures are probability distribution histograms borrowed from a computer graphics technique, ray-tracing. In this method, a ray is initiated within a molecule bound by its solvent accessible surface. Propagation of a ray trace inside of the triangulated solvent accessible surface is recorded as probability distribution histograms. The histograms for query and any other molecule can be easily compared using the following metrics:

$$\begin{array}{lll} L_{1}^{1D} & = & \;\underset{i}{\sum }|H_{i}^{1}-H_{i}^{2}|\\ L_{1}^{2D} & = & \;\underset{i}{\sum }\underset{j}{\sum }|H_{i,j}^{1}-H_{i,j}^{2}| \end{array}$$

where 1D represents the probability distribution of ray-trace lengths only while 2D represents ray-trace lengths in combination with additional molecular property such as electrostatic potential. Shape signature encodes shape, molecular size and surface charge distribution of a molecule and can be utilized to compare the histogram of a query molecule with the pre-generated histograms of small molecule libraries. The utility of shape signatures as a virtual screening approach has been demonstrated in several studies (Nagarajan et al., 2005; Wang et al., 2006; Hartman et al., 2009; Ai et al., 2014; Werner et al., 2014). As shape signature based similarity comparisons are fast and do not require the alignment of molecules, they are capable of screening millions of molecules in a short time. In addition to shape similarity, shape signatures also allow shape complementarity comparisons against a receptor binding pocket. Although shape similarity calculations with shape signature have been effectively used in many inhibitor discovery efforts, the high number of false positives is a concern especially for large and complex queries. To cope with these drawbacks, a few modifications to the original methods were reported. These include fragment-based shape signature (FBSS) (Zauhar et al., 2013) and inner distance shape signature (IDSS) (Liu et al., 2009, 2012). FBSS involves the generation and comparison of shape signatures for fragments in the molecules. IDSS utilizes inner distance which is the shortest path between landmark points within the molecular shape. IDSS has been shown to be especially useful in case of flexible molecules as it is insensitive to shape deformation of flexible molecules.

Several methods employed local surface shape similarity to align and estimate the similarity between molecules. One such method applied subgraph isomorphism to molecular surface comparison (Cosgrove et al., 2000). In this method, molecular surface was represented by patches of the same shape. Alignment between two molecules was obtained by using a clique-detection algorithm to obtain overlapping patches. Quadratic shape descriptors (Goldman and Wipke, 2000) exploited a similar concept where molecular surface was divided into a series of patches. Each patch was represented by geometrically invariant descriptors such as the normal, the shape index and the principle curvatures which were then used to identify similar patches. SURFCOMP (Hofbauer et al., 2004) further applied several filters such as surrounding shape and physicochemical properties to identify corresponding patches on surfaces of two molecules (Table 3).

**Table 3:** An overview and availability of a few surface-based shape comparison methods.

**Method**	**Description**	**Availability**	**References**
SURFCOMP	Molecular surface is divided into patches and corresponding patches are identified using geometrically invariant descriptors and physicochemical properties.	Available on request.	Hofbauer et al., 2004
ParaFit	Performs 3D superposition and surface property comparison. Electronic surface properties are calculated using ParaSurf program. Spherical harmonics expansion coefficients of molecular surface are used.	Developed by CEPOS *in silico* Ltd. Commercial or Academic license can be obtained at http://www.ceposinsilico.de/	Mavridis et al., 2007
SHeMS	Uses spherical harmonics description of shape. Weights of spherical harmonics expansion coefficients are optimized using a genetic algorithm.	Developed by Shanghai Key Laboratory of New Drug Design, East China University of Science & Technology, Shanghai, China. Obtained by contacting Prof. Honglin Li at http://lilab.ecust.edu.cn/home/resource.html	Cai et al., 2012
HPCC	Combined spherical harmonics shape comparison with pharmacophoric features. Tanimoto similarity coefficients for shape and chemical similarity are added to evaluate similarity between two molecules.	Developed by Harmonic Pharma. May be available from https://www.harmonicpharma.com/oncology/	Karaboga et al., 2013
3DZD	Uses 3D Zernike descriptors which are extension of spherical harmonics. Rotation translation invariant.	Developed by Kihara Bioinformatics laboratory at Purdue University, USA. Several implementations of 3DZD are available either as standalone program or web-server at http://kiharalab.org/contact.php	Sael et al., 2008a, Venkatraman et al., 2009a

Spherical harmonics (SH) based representations which are expansion of SH functions also allow quantitative description of molecular shapes (Max and Getzoff, 1988). In this representation, shapes are expressed as functions on a unit sphere. Each point on a unit sphere surface is described by its spherical coordinates (*r*,θ,ϕ) and setting *f* (θ,ϕ) = *r*, where r is a radial function encoding the distance of surface points from a chosen origin. This function can be determined by deriving an expansion of SH basis function given by:

$$r\left(\theta ,\phi \right)=\overset{L}{\underset{l=0}{\sum }}\overset{l}{\underset{m=-l}{\sum }}c_{l,m}Y_{l}^{m}\left(\theta ,\phi \right)$$

where $Y_{l}^{m}\left(\theta ,\;\phi \right)$ is the SH basis function for degree *l* and order *m*. *c*_*l, m*_ are coefficients of SH function. L is the chosen limit to get desired resolution of the surface. The number of terms in the function depends upon this limit as a value of L, which yields (L+1)^2^ terms. In general, SH are not rotation translation invariant as magnitude of *c*_*l, m*_ change based on the rotation of *r*(θ, ϕ). Hence, prior alignment is necessary before comparing the shape of molecules. Efforts were also made to make SH rotation translation invariant (Kazhdan et al., 2003; Mak et al., 2008), however, these modifications increase the number of terms thereby increasing the complexity of SH.

About two decades ago, it was shown that SH functions could be applied to estimate the 3D molecular similarity between two macromolecules (Ritchie and Kemp, 1999). Since then, it has been successfully applied in virtual screening (Cai et al., 2002; Mavridis et al., 2007), protein structure comparisons (Tao et al., 2005; Gramada and Bourne, 2006), protein-ligand docking (Ritchie and Kemp, 2000; Lin and Clark, 2005; Yamagishi et al., 2006), binding pocket similarity comparison (Morris et al., 2005) etc. Additionally, several groups utilized variations of SH to compare the shapes of small molecules. The first implementation of SH to compare shapes of small molecules opened the way for many applications ranging from virtual screening to quantitative structure-activity relationship (QSAR) model building (Lin and Clark, 2005). SpotLight program utilizes SH to superpose and classify small molecules (Mavridis et al., 2007). To enable high throughput virtual screening, the vector interpretation of SH coefficients was used to construct rotation translation invariant fingerprints (RIFs) which were compared using a distance score (Mavridis et al., 2007). In this method, rotational invariance was gained by binning together the SH coefficients of the same order. This method was later developed as ParaFit (http://www.ceposinsilico.de) (Table 3). In another study, SH based molecular surface was decomposed and the norm of decomposition coefficients were used to describe the molecular shape (Wang et al., 2011). Norms of decomposition coefficients are partially rotation translation invariant enabling large scale comparison. The performance of this method was retrospectively demonstrated and was also prospectively applied in the discovery of cyclooxygenase-1 and cyclooxygenase-2 inhibitors. SHeMS method utilizes genetic algorithm to optimize the weights of SH expansion coefficients for a reference set (Cai et al., 2012). Through optimization of weights, SHeMS demonstrated improved performance over original SH implementation and USR method. To facilitate measurement of similarity between sets of compounds, many shape similarity methods were complemented with physicochemical properties. Harmonic pharma chemistry coefficient (HPCC) method combined SH shape representation with pharmacophoric features (Karaboga et al., 2013). In HPCC method, SH surfaces are discretized as triangle meshes which are assigned pharmacophoric features. Tanimoto similarity for both shape and pharmacophore features is calculated separately between query and test molecules. A combo score is finally calculated by adding Tanimoto scores for shape and chemical overlay. HPCC method demonstrated improved performance for the combo approach over utilizing the shape alone.

**Figure 4:**
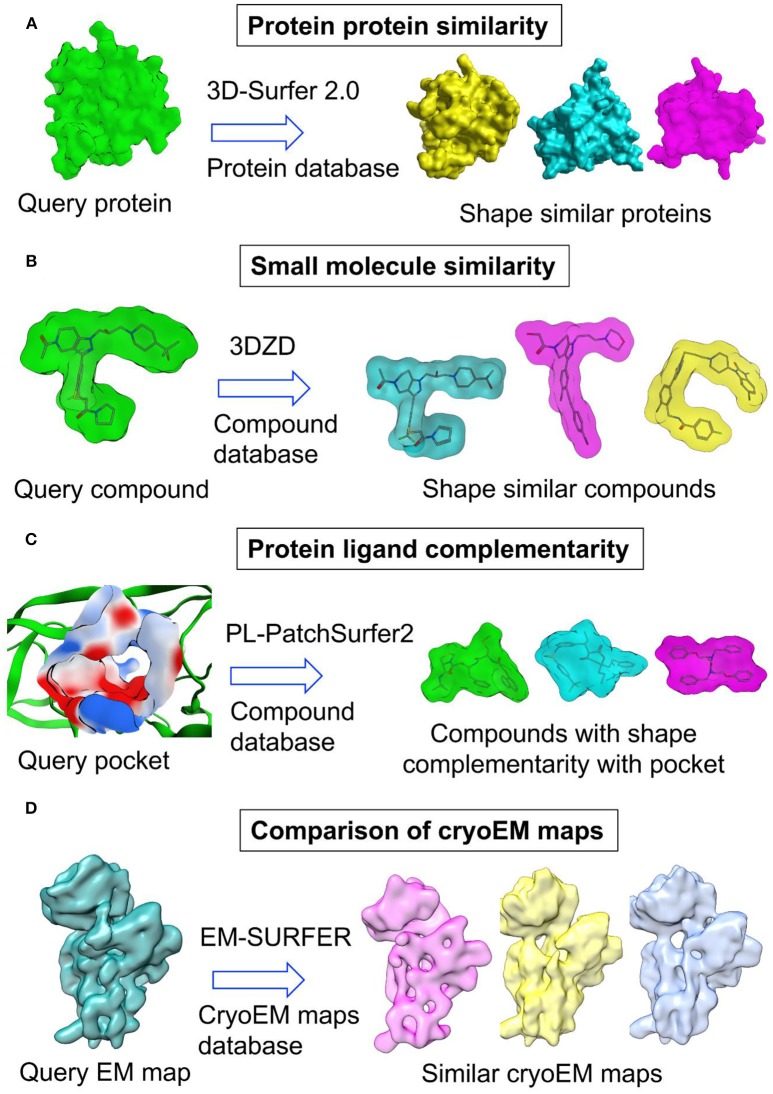
Application of 3D Zernike descriptors in **(A)** protein protein similarity **(B)** small molecule similarity **(C)** protein ligand complementarity and **(D)** comparison of cryoEM maps.

In several studies, 3D-Zernike descriptors (3DZD) (Novotni and Klein, 2003), which are the extension of SH were employed to compare the shapes of molecules and cryoEM maps (Figure 4 and Table 3). 3DZD differs from SH in terms of their mathematical description. 3DZD can model molecular shape precisely as compared to SH which can only model single valued or star-shape surfaces. They are rotation translation invariant, whereas SH depends on the orientation of the molecule. Although rotation translation invariant SH descriptors have been developed (Kazhdan et al., 2003), the number of terms are much higher in SH descriptors. 3DZD is also suitable to represent other properties on molecular surfaces such as hydrophobicity and electrostatic potential (Sael et al., 2008a). In the drug discovery area, 3DZD was initially applied to compare shapes of protein molecules (Sael et al., 2008b; Figure 4A). Later, the concept was extended to measuring shape similarity and small molecules (Venkatraman et al., 2009a) and between binding pockets (Kihara et al., 2009; Venkatraman et al., 2009b; Figures 4B,C). In 3DZD method, 3D Zernike function is described as:

$$Z_{nl}^{m}\left(r,\theta ,\phi \right)=R_{nl}\left(r\right)Y_{l}^{m}\left(\theta ,\phi \right)$$

where $Y_{l}^{m}\left(\theta ,\;\phi \right)$ is the SH basis function while *R*_*nl*_(*r*) is the radial function. Zernike moments are calculated using the following equation:

$$F_{nl}^{m}=\frac{3}{4\pi }\int f\left(x\right)\bar{Z_{nl}^{m}\left(x\right)}dx$$

As Zernike moments are not rotationally invariant, so to make them rotation translation invariant, they are expressed as norm $F_{nl}^{m}$ which is known as 3DZD. Shape similarity between two molecules based on 3DZD is compared using the following metrics:

$$\begin{array}{l} Euclideandistance=\sqrt{\overset{n}{\underset{i=1}{\sum }}{\left(X_{i}-Y_{i}\right)}^{2}}\in \left[0,\infty \right]\\ Pearsonr=\frac{n\sum X_{i}Y_{i}-\sum X_{i}\sum Y_{i}}{\sqrt{n\sum {X_{i}}^{2}-{\left(\sum X_{i}\right)}^{2}}\sqrt{n\sum {Y_{i}}^{2}-{\left(\sum Y_{i}\right)}^{2}}}\in \left[-1,1\right]\\ Manhattandistance=\frac{1}{1+\frac{\sum_{i=1}^{n}|X_{i}-Y_{i}|}{N}}\in \left[0,1\right] \end{array}$$

Ligand 3D shape similarity comparison using 3DZD is fast and rotation translation invariant. As no alignment step is required for comparison, it can be utilized as a virtual screening tool to filter a database of compounds based on shape similarity with a query molecule.

Overall, surface-based shape similarity methods present attractive options for comparing the shapes of small molecules and macromolecules. They were quite successful in estimating the global and local similarities between macromolecules. However, most of these methods are still in infancy as far as small molecule shape comparison is concerned. Several reasons may have contributed to the lack of interest from researchers in accepting these methods as small molecule shape comparison tools. Surface-based methods such as SH and 3DZD are mathematically complex and involve inclusion of many terms to fully capture the shape of a molecule. Moreover, they are slow in comparison to atomic distance-based shape description and comparison methods while their accuracy in retrieving compounds similar in shape to a query does not match Gaussian overlay-based shape similarity methods. Further, while these methods capture very well the global shape of a molecule, the local shape similarity is not represented comprehensively which is very critical in comparing the shapes of small molecules. However, these methods present several new areas of shape comparison such as comparing shape of ligands with that of binding pockets which may be of immense utility for structure-based design.

### Other shape similarity approaches

There are many other approaches of shape representation and methods of similarity measurement in addition to these described above. Another way of representing molecular shape is to use molecular descriptors. Several shape-based descriptors have been traditionally used to compare small molecules and develop QSAR models. These descriptors mostly represent shape implicitly with other properties such as size, symmetry and atom distribution. These include Weighted Holistic Invariant Molecular (WHIM) descriptors of shape (Gramatica, 2006), shape indices, descriptors for moments of the distribution of molecular volume (Mansfield et al., 2002). Most of molecular descriptors are alignment independent, however, some such as moments of the distribution of molecular volume require superposition of molecules. Comparative Molecular-Field Analysis (CoMFA) (Cramer et al., 1988) is a widely used technique to develop QSAR models and understand SAR for a series of compounds. CoMFA compares a set of molecules by placing them on a grid and calculating potential energy fields. The differences and similarities between molecules are then correlated with differences and similarities in their biological activities. As CoMFA requires molecules to be pre-aligned, the 3D shape similarity of molecules can be obtained based on potential energy fields. A modification of CoMFA approach, Comparative Molecular Moment Analysis (CoMMA) calculates geometric moments from the center of mass, center of charge and center of dipole of a molecule (Silverman and Platt, 1996). However, superposition of molecules is not required in this approach. Shape of the molecules can also be inferred from structural descriptors such as molecular quantum numbers (MQNs) (Nguyen et al., 2009; van Deursen et al., 2010). The MQN represents counts for 42 structural features such as atom, ring and bond types, polar groups and topology. MQN system has been used to effectively classify and visualize large libraries of organic molecules such as ZINC, GDB, and PubChem.

Volumetric aligned molecular shapes (VAMS) method (Koes and Camacho, 2014) uses data structures to represent and compare shapes of 3D molecules. It applies inclusive and exclusive shape constraints to estimate the similarity in shapes of 3D molecules. In VAMS method, the shape of a molecule is represented by solvent-excluded volume calculated from its heavy atoms using a water probe of radius 1.4 Å. Volume is discretized on a grid of 0.5 Å resolution where each point on the grid represents a Voxel or 3D pixel. An oct-tree data structure is used to store voxelized volume. This method requires all the shapes to be pre-aligned to a standard reference coordinates. The conformations of the molecule are aligned using the moment of inertia of heavy atoms. Voxelized shapes are compared using Tanimoto similarity (Rogers and Tanimoto, 1960) where the ratio of number of voxels common in two shapes and number of voxels present in either of the shapes is measured. The performance of VAMS method as a standalone virtual screening tool is not better than many other shape similarity methods, e.g., ROCS, however, VAMS is reasonably fast and could perform a million shape comparisons in about 10 s. Hence, it may be used as a pre-filtering tool for other shape similarity methods. Fragment oriented molecular shape (FOMS) is the extension of VAMS method, where shapes are aligned using fragments (Hain et al., 2016).

## Application of shape similarity methods in drug discovery

### Application in virtual screening

Shape similarity attempts to quantify the resemblance between two molecules utilizing several descriptions of molecular shape as described previously. This approach has been successfully utilized as a virtual screening tool to identify molecules similar to a given query from the library of chemicals. Several retrospective studies have been published demonstrating the utility of shape based similarity methods over 2D and other 3D similarity methods (Nagarajan et al., 2005; Renner and Schneider, 2006; Ballester et al., 2009; Giganti et al., 2010; Venkatraman et al., 2010; Ballester, 2011; Hu et al., 2012, 2016). Several studies also presented computational approaches to improve the performance and efficiency of shape comparison methods. One study recommended the selection of a suitable query and incorporation of chemical information such as pharmacophoric features of the query molecule to improve the performance of shape-based virtual screening (Kirchmair et al., 2009). Another study demonstrated that the application of a machine learning method, Support Vector Machine (SVM), to shape comparisons can significantly improve virtual screening efficiency (Sato et al., 2012). The need of automation was further suggested specially to carry out multiple query searches which ensure a diverse hit list (Kalászi et al., 2014).

Apart from retrospective tests, many prospective applications of shape similarity have been published in the literature. In numerous studies, it was employed as the only virtual screening approach to filter and prioritize compounds from a large library to a number small enough for biological testing (Rush et al., 2005; Boström et al., 2007; Freitas et al., 2008; Ballester et al., 2010, 2012; Kumar et al., 2012; Vasudevan et al., 2012; Sun et al., 2013; Hoeger et al., 2014; Patil et al., 2014; Temml et al., 2014; Chen et al., 2016; Bassetto et al., 2017). Among these studies, the shape based identification of a compound active on colon cancer cell line is quite interesting (Patil et al., 2014). This study employed USR to screen a database of approved drugs. The top virtual screening hit displayed dose dependent inhibition of a colon cancer cell line. This study not only repurposed a known drug but also demonstrated the applicability of shape similarity methods for phenotypic screens, e.g., anti-bacterial or anti-fungal drug discovery where molecular target is often unknown. This is especially important considering the fact that most approved drugs come from phenotypic screens (Swinney and Anthony, 2011). In other investigations, it was combined with other ligand-based virtual screening methods or structure based approaches such as molecular docking. Among ligand-based approaches, shape similarity was frequently used in combination with electrostatic similarity. As electrostatic comparison between two small molecules requires precise alignment between them, shape matching was first performed and then followed by the electrostatic potential similarity calculations. This hierarchical combination was utilized to discover a wide variety of binders including enzyme inhibitors (Hevener et al., 2011), mRNA binders (Kaoud et al., 2012), chemical probes (Naylor et al., 2009), protein-protein interaction inhibitors (Boström et al., 2013), SUMO activating enzyme 1 inhibitors (Kumar et al., 2016), and Aurora kinase A inhibitors (Kong et al., 2018).

Although shape-based approaches demonstrated considerable success in ligand-based virtual screening studies, the true potential of the method was realized when it was combined with structure based methods in a hierarchical manner or in a parallel manner. To effectively use shape based virtual screening, several groups employed hierarchical virtual screening (Kumar and Zhang, 2015) where it was coupled with molecular docking. As shape matching calculations are comparatively faster than structure based virtual screening methods, it is generally used during initials steps of a hierarchical virtual screening protocol. This hierarchical combination of shape similarity with molecular docking has been successfully employed in the discovery of type II dehydroquinase inhibitors (Ballester et al., 2012) and that of MDM2 inhibitors (Houston et al., 2015), 11β-hydroxysteroid dehydrogenase 1 inhibitors (Xia et al., 2011), PPARγ partial agonists (Vidović et al., 2011), inhibitors of chemokine receptor 5 (CCR5)-N terminus binding to gp120 protein (Acharya et al., 2011), Grb7-based antitumor agents (Ambaye et al., 2013), fungal trihydroxynaphthalene reductase inhibitors (Brunskole Švegelj et al., 2011), non-steroidal FXR ligands (Fu et al., 2012; Wang et al., 2015), novel SIRT3 scaffolds (Salo et al., 2013), protein kinase CK2 inhibitors (Sun et al., 2013), SUMO conjugating enzyme inhibitors (Kumar et al., 2014a), and chemokine receptor type 4 inhibitors (Das et al., 2015). Combination of shape similarity methods with structure-based methods such as docking provide several advantages. Ultrafast shape comparison methods such as USR can very quickly filter large libraries for compounds that are similarly shaped as known inhibitors. Hence, the time required for docking could be drastically reduced by eliminating compounds that doesn't fit in the binding pocket. Moreover, in case of some proteins the inhibitor activity is driven by key moieties in compounds, e.g., metal binding groups in case of metalloproteins, reactive functional groups in cysteine proteases, hinge binding groups in kinases etc. In these scenarios, docking will help in the prioritization of compounds based on the interactions they make with the binding pocket. Sometimes the difference in shape similarity scores for compounds is very small and it is challenging to cherry pick for biological assay. Here, docking of shape similarity hits could also help in the prioritization of compounds for purchase or chemical synthesis. However, the combination of shape similarity with molecular docking is not always advantageous especially for proteins with highly flexible binding pockets, multiple pocket conformations or homology models where accurate docking is challenging. A virtual screening scheme where USR hits were re-ranked using Autodock-Vina score produced no active hits as docking was performed in a quite different pocket conformation (Hoeger et al., 2014). In another study, shape-based virtual screening alone produced better hit rates than hierarchical combination of shape similarity and docking methods (Ballester et al., 2012). In numerous studies, shape similarity calculations along with molecular docking were complemented with other approaches such as 2D similarity search, pharmacophore modeling, electrostatic potential matching, machine learning and MM-PBSA method (Mochalkin et al., 2009; Alcaro et al., 2013; Poongavanam and Kongsted, 2013; Wiggers et al., 2013; Hamza et al., 2014a; Kumar et al., 2014b; Pala et al., 2014; Feng et al., 2015; Corso et al., 2016; Mangiatordi et al., 2017; Xia et al., 2017). The use of different virtual screening approaches in parallel has been previously suggested as different methods tend to identify different set of compounds and virtual screening hit rates could be improved by employing them in parallel manner (Sheridan and Kearsley, 2002). In parallel virtual screening, several methods are run independently and the top hits from each method is selected. Parallel combination of various ligand and structure based methods with shape similarity approaches was found to be productive especially in case of challenging targets (Swann et al., 2011; Langdon et al., 2013; Hoeger et al., 2014). A parallel virtual screening to identify inhibitors of PRL-3 employing several ligand and structure-based methods against the same screening library produced contrasting hit rates for different approaches (Hoeger et al., 2014). Many prospective applications suggest the utility of hierarchical or parallel combination of shape similarity approaches with other ligand and structure-based methods. However, no benchmark study demonstrating their utility has been published. A systematic study will help researchers to identify areas where the combination of several approaches will be better than employing shape based virtual screening methods alone.

One application of shape similarity methods is to hop from one chemical scaffold to another in order to improve the potency, selectivity, physicochemical properties and to create novel intellectual property positions (Hu et al., 2017). Shape similarity methods are capable of identifying several scaffolds which are structurally different from the query compounds and each scaffold may be pursued separately. Scaffold hopping is highly effective in rescuing the problematic leads that cannot be pursued further due to problems in selectivity, pharmacology and pharmacokinetics. Both atomic distance-based and Gaussian-overlay shape similarity methods can effectively perform scaffold hopping as exemplified from several prospective studies. Among the first prospective application of shape similarity based methods in scaffold hopping, small molecule inhibitors of ZipA-FtsZ protein-protein interaction were identified (Rush et al., 2005). Some recent scaffold hopping applications include the identification of inhibitors of arylamine *N*-acetyltransferases (Ballester et al., 2010), type II dehydroquinase inhibitors (Ballester et al., 2012) sumoylation enzymes (Kumar et al., 2014b, 2016), anti-tubercular agents (Hamza et al., 2014b; Wavhale et al., 2017), anti-tumor agents (Ge et al., 2014), 11βHSD1 inhibitors (Shave et al., 2015), leucine zipper kinase inhibitors (Patel et al., 2015), kynurenine 3-monooxygenase inhibitors (Shave et al., 2018), and partial agonist of inositol trisphosphate receptor (Vasudevan et al., 2014). In addition to prospective application, rigorous benchmarking of shape similarity methods for their scaffold hopping capabilities is important. However, systematic benchmarking is challenging due to disagreement on the definition of scaffold. In one retrospective study, the scaffold hopping potential of atomic distance-based shape similarity method USRCAT has been demonstrated utilizing DUD-E dataset (Schreyer and Blundell, 2012). For the tested benchmark dataset, USRCAT was capable of identifying structurally dissimilar active hits that could not be retrieved by utilizing topological similarities. Shape similarity was also used to repurpose existing drugs for previously unknown activity (Vasudevan et al., 2012). Another application is *in silico* target fishing or the identification of protein targets of orphan chemical compounds. In one recent research, the target of anti-fungal macrocycle amidinoureas was identified following a shape similarity screening (Maccari et al., 2017). The representative structure from a series of macrocycle amidinoureas was used as a query to obtain most similar crystallographic ligand from all solved crystal structures. A prioritized list of targets based on similarity score and subsequent docking and enzymatic assay revealed *Trichoderma viride* chitinase as target of this class of compounds. Along the same line, retrospective studies showed that the combination of molecular shape and chemical structure similarity can reliably achieve biological target prediction (Abdulhameed et al., 2012; Gfeller et al., 2013). Additionally, shape similarity comparison based on spherical harmonics surface representation has been demonstrated that it can be used to predict drug promiscuity (Perez-Nueno et al., 2011). Furthermore, shape similarity comparisons could also be used to predict subtype selectivity of ligands (Kuang et al., 2016).

One important application of shape similarity methods in drug discovery is the clustering of known inhibitors of a protein target. As the performance of most shape-based methods highly depend on the selection of right query for the virtual screening (Kirchmair et al., 2009), special attention was paid toward the development of methods dealing with this problem. It has been reported that clustering of known inhibitors based on their shapes could help the identification of optimal query for virtual screening (Pérez-Nueno and Ritchie, 2011). Clustering of spherical harmonics-based consensus shapes assisted in the identification of ligands that bind to different regions in the binding pocket of some protein targets such as CCR5 (Pérez-Nueno et al., 2008). Further, the clustering of molecular shapes also helped in the identification of promiscuous protein targets and ligands (Pérez-Nueno and Ritchie, 2011). Selection and use of high quality compound libraries is an important aspect of high throughput screening (HTS). However, testing a large number of compounds is not economically viable. *In silico*, mostly 2D similarity based, methods are commonly employed to generate a subset or focused set for HTS (Huggins et al., 2011; Dandapani et al., 2012). The limitation with 2D similarity methods is that they ignore inherent property such as the shape of a molecule. Use of shape-based clustering of large compound libraries for creating quality HTS library present several advantages. Clustering of molecular libraries based on atomic distance-based methods such as USR can achieve similar or significantly better computational efficiency as 2D fingerprint-based methods. Moreover, it will ensure maximum diversity with less number of compounds in HTS library.

Apart from employing ligand 3D shape similarity as a virtual screening method, several groups adopted it to improve the performance of other virtual screening methods. Molecular docking is one such method widely used in drug discovery. Although there has been significant progress in the development of molecular docking methods, challenges still remain both in sampling and scoring of binding poses within protein binding pockets. In the last few years, several methods were developed that utilized ligand 3D shape similarity to improve both sampling and scoring performance of molecular docking. The shape overlap with known crystallographic ligands for the target protein was utilized to guide ligand conformational sampling toward critical regions of protein binding site (Wu and Vieth, 2004). Other methods used shape similarity based alignment for the selection of reliable poses among many docking generated poses (Fukunishi and Nakamura, 2008, 2012; Anighoro and Bajorath, 2016; Kumar and Zhang, 2016a). Ligand 3D shape similarity was also a key component of many pose prediction methods where shape similarity with existing ligand bound crystal structures was utilized to predict binding poses of unknown ligands (Kelley et al., 2015; Huang et al., 2016; Kumar and Zhang, 2016b,c). Several of these methods demonstrated excellent retrospective and prospective performance. Moreover, shape similarity also facilitated the improvement in scoring and rank-ordering performance of a docking method. Several methods have reported improved virtual screening performance of a docking method when shape overlap with crystallographic ligands was employed to select the best binding pose of ligands in a screening library (Roy et al., 2015; Anighoro and Bajorath, 2016). Consideration of protein flexibility in molecular docking is a challenging problem and several methods have been developed to tackle it (B-Rao et al., 2009). Among these, receptor ensemble based methods demonstrated reasonable performance (Bottegoni et al., 2011) where the receptor ensemble is selected either from many crystallographic structures or from those generated by *in silico* methods such as molecular dynamics simulation. It has been shown previously that the selection of receptor ensemble based on binding pocket shape similarity is an effective way of considering receptor flexibility in molecular docking (Osguthorpe et al., 2012). Further, one method suggested utilizing a single suitable receptor for each ligand in a screening library instead of docking all compounds to multiple receptor structures (Kumar and Zhang, 2018). It was also shown that single suitable receptor selection based on ligand 3D shape similarity is superior to 2D similarity based selection.

### Applications in protein structure comparison

Evaluation of structural similarity between protein structures has many applications including but not limited to classification of protein structures, evolutionary relationship between protein structures, identification of templates for homology modeling, functional annotation, protein-protein interactions etc. Conventional methods for protein structure comparison are based on the alignment of protein atoms or residues. These methods require extensive rotational and translational sampling thereby limiting their utility for large scale protein structure comparisons. Several methods have been developed that utilize shape similarity to detect global or local similarity between protein structures. Classification of these methods also follows the previously described classification including Gaussian overlay based methods, surface-based methods using spherical harmonic descriptors, 3D Zernike descriptors etc. Among these, surface-based methods were developed previously to measure similarity between protein structures. Only later they were applied to the small molecule area. Several methods of protein structure comparison employed SH to represent shapes of protein structures (Tao et al., 2005; Gramada and Bourne, 2006; Konarev et al., 2016). Like SH, 3D Zernike based moments are also suitable to compare shapes of protein structures (Sael et al., 2008b; Figure 4A). Not only they were suitable to estimate the similarity between two proteins but also their rotation-translation invariant nature allows fast real-time search of similar proteins in structural databases such as PDB (La et al., 2009; Kihara et al., 2011; Xiong et al., 2014). A Gaussian mixture model based protein shape similarity method (Kawabata, 2008) also allows large scale comparisons of proteins with data from PDB and EMDB. This method has been implemented as Omokage search in PDB Japan (Suzuki et al., 2016; Kinjo et al., 2017). The server compares global shapes of proteins and results are obtained reasonably fast within 1 min after submission of a query. Large scale comparison of protein structures based on shape is useful in functional annotation, selection of templates for comparative modeling etc. An application of shape comparison method to protein classification has also been reported (Daras et al., 2006).

One important application of shape matching is the evaluation of similarity between protein binding pockets. This field is especially interesting as sequence and structural alignments are often not useful when comparing binding pockets of proteins with different folds. As protein binding pockets are much more conserved than protein structures (Gao and Skolnick, 2013), a reliable comparison between protein binding pockets is crucial for predicting protein functions, polypharmacology of ligands and for drug repurposing. Numerous methods based on distinct structural representations as described previously were developed in the last decade. One such method employed spherical harmonics to represent and compare the shapes of protein binding pockets (Morris et al., 2005). This method was later extended to compare the shape of protein binding pockets with that of binding ligands (Kahraman et al., 2007). PocketMatch compares two binding pockets based on the sorted list of distances that captured chemical nature and 3D shape of the binding pocket (Yeturu and Chandra, 2008). Another method based on property-encoded shape distributions (PESD) combines the concept of shape distributions with the chemical environment of the binding pocket surface to effectively capture binding pocket similarities (Das et al., 2009). Pocket-Surfer utilizes pseudo-Zernike descriptors and 3D Zernike descriptors to represent and compare properties and 3D shapes of binding pockets (Chikhi et al., 2010). An extension of this method, Patch-Surfer searches local similarity by representing a binding pocket as amalgamation of segmented surface patches which are described by properties such as shape, electrostatic potential, concaveness and hydrophobicity (Sael and Kihara, 2012). Similarity between protein cavities was also measured by representing the pockets by pharmacophoric grid points and aligning them by optimizing their volume overlap (Desaphy et al., 2012).

Concept of pocket similarity was also extended to complementarity between binding pockets and ligands. This gave rise to a new virtual screening methodology based on shape complementarity between binding pockets and ligands. PL-Patch-Surfer2 program evaluates the compatibility between ligand and binding pocket by measuring the complementarity between ligand surface and local surface patches in the binding pocket (Shin et al., 2016a,b; Figure 4C). The program utilizes 3DZD to represent molecular shape while physicochemical properties are also mapped onto the surface. The method was evaluated on benchmark datasets and revealed better performance than two docking programs. Spherical harmonics expansion coefficients have also been employed in the approximation and comparison of binding pockets and ligand surfaces (Cai et al., 2002). The complementarity was demonstrated utilizing 35 protein-ligand complexes. Elekit adopted shape and electrostatic complementarity concept to discover small molecule inhibitors of protein-protein interactions (Voet et al., 2013). Elekit assesses the similarity between small molecules and protein ligands of a receptor protein based on the electrostatic potential values stored on a 3D grid.

### Applications in fitting of atomic models into cryo-electron microscopy maps

Recent developments in cryo-electron microscopy (cryo-EM) has helped researchers to overcome resolution barrier and provide structural and mechanistic insights into structures of difficult proteins and large protein assemblies. Most of these improvements came from the advances in sample preparation, electron detector technologies, improved microscope and computational data processing. Computational methods played an important part in particle picking, particle reconstruction, building and fitting of structures into cryo-EM maps. In recent years, several methods were developed to improve building, fitting and refinement of protein structures in cryo-EM maps (Esquivel-Rodríguez and Kihara, 2013). Among these methods, a few methods employed shape similarity to fit atomic structures of protein subunits into the cryo-EM maps of multi-subunit proteins. One method, Gaussian Mixture macromolecule FITting (gmfit), utilizes Gaussian mixture models (GMM) to represent the shape of cryo-EM maps and atomic models (Kawabata, 2008). GMMs are probability distribution functions obtained by joining many 3D Gaussian functions. Initially, both the cryo-EM map and atomic models are first converted into GMM followed by the fitting of a single subunit GMM into the GMM of protein complex using random and gradient based local search. Finally, the fit between atomic models and cryo-EM map is obtained based on the position and orientation of GMM. This method is reasonably fast and can fit multiple subunits with reasonable accuracy. PDB Japan (https://pdbj.org) has implemented this method in its EM navigator utility to provide shape based structural similarity search against protein databases (Kinjo et al., 2017). Another method adopted a surface-based approach where 3DZD was used to represent and compare isosurface derived from low resolution cryo-EM maps of protein structures (Sael and Kihara, 2010; Figure 4D). It was demonstrated that 3DZD can distinguish proteins of different folds even at low resolution of 15 Å. A web-based platform for comparing cryo-EM maps was also developed by the same group (Esquivel-Rodríguez et al., 2015; Han et al., 2017). A similar method utilized 3D Zernike moments to search a database of protein structures for matching protein structures to a cryo-EM map (Yin and Dokholyan, 2011). EMLZerD method also utilized 3DZD to fit multiple structures in a cryo-EM map (Esquivel-Rodríguez and Kihara, 2012). The method generates hundreds of putative configurations of subunit arrangement using a protein-protein docking method. These configurations were later compared with a cryo-EM map using 3DZD and Euclidean distance. The biggest advantage of 3D Zernike moments methods is that they are rotation translation invariant and no computational expensive step of rigid body or flexible structural alignment is required. Moreover, these methods enable screening of proteins from structural databases such as PDB to find out models that can fit into a cryo-EM map.

## Conclusion and future directions

3D shape similarity methods have contributed immensely to the overall acceptance of the computational virtual screening methods in drug discovery. Most shape similarity methods for shape comparison of small molecules and macromolecules took inspiration from the approaches developed to compare the shapes of 3D objects in computational geometry field. Several approaches were developed ranging from extremely fast atom distance-based methods to comparatively slower mathematically complex methods such as SH and 3DZD. Among all the 3D shape comparison methods, atomic distance-based and Gaussian overlay-based methods are the most widely used. These approaches possess several advantages over surface-based methods. Atomic distance-based methods present an extremely fast way of quickly comparing the shapes of small molecules. This has facilitated the screening of very large libraries of millions of compounds within a few seconds. Moreover, screening large libraries increased the probability of finding novel chemical scaffolds. Furthermore, as most of these methods depend on shape rather than the underlying chemical structure, scaffold hopping can be conveniently achieved. Another possible application of these fast shape similarity evaluation methods would be the clustering of large chemical space to generate quality shape diverse HTS screening libraries. Although Gaussian overlay-based methods are slower than atomic-distance based methods, they are fast enough to allow high throughput virtual screening. GPU implementations of these methods is not very difficult as exemplified by the development of several GPU compatible programs such as FastROCS, PAPER, gWEGA etc. resulting in further increase in the processing speeds. Another advantage with Gaussian-based methods is that they allow visualization as they require alignment of molecules prior to shape similarity calculations. Visualization is helpful in understanding the features responsible for biological activity and critical for the optimization of a molecule especially for the molecules with low structural similarity with query compound. However, a suboptimal alignment can lead to errors in volume overlap calculations and thereby affecting similarity scores and visualization. As alignment is the key component of Gaussian overlay methods, efforts should be focused toward improving molecular alignment. Some of these methods employ chemical features to refine global overlays. As alignment is global optimization problem, molecular alignment could also be improved by employing fast local optimization methods. Both atomic distance-based and Gaussian overlay-based shape similarity methods handle ligand flexibility by employing the conformational ensemble. The performance thus indirectly depends upon conformation generation methods. Current state-of-the-art conformation generation methods still struggle to generate near-native conformations of ligands such as peptidomimetics, macrocycles etc. Development of novel conformation generation approaches utilizing knowledge from experimental databases such as CSD and PDB will steer improvement in performance of shape-based virtual screening approaches. Surface based methods such as SH expansion coefficients and 3DZD are suitable for comparing macromolecules and atomic models with electron density maps, however, comparatively less efforts have been made toward utilizing them in small molecule area. One advantage with surface-based methods is that the protein ligand complementarity search is possible by comparing enclosed shapes of binding pockets and ligands. This will be handy in cases where ligand-based virtual screening methods could not be used due to the lack of active compounds. Finally, shape-based similarity could be used in combination with other ligand and structure-based approaches either in hierarchical or parallel manner to improve hit rate especially for difficult targets.

## Author contributions

All authors listed have made a substantial, direct and intellectual contribution to the work, and approved it for publication.

### Conflict of interest statement

The authors declare that the research was conducted in the absence of any commercial or financial relationships that could be construed as a potential conflict of interest. The reviewer XL and handling Editor declared their shared affiliation.
